# Analysis and design of ultra-wideband PRGW hybrid coupler using PEC/PMC waveguide model

**DOI:** 10.1038/s41598-022-18343-0

**Published:** 2022-08-20

**Authors:** Zahra Mousavirazi, Mohamed Mamdouh M. Ali, Hassan Naseri Gheisanab, Tayeb A. Denidni

**Affiliations:** 1 Institut National de la Recherche Scientifique (INRS), Montreal, H5A 1K6 Canada; 2 Department of Electrical Engineering Faculty of Engineering, Assiut University, Assiut, Egypt

**Keywords:** Engineering, Electrical and electronic engineering

## Abstract

In this paper, a new method to facilitate the design of Printed Ridge Gap Waveguide (PRGW) structures is introduced. One of the main difficulties in designing such structures is related to their simulation process which is really time and energy-consuming. Therefore, a suitable boundary condition is considered to bring about the primary structure without involving the bed of nails or mushroom unit cells. Using this technique, a wideband PRGW 3 dB hybrid double-box coupler is designed to serve in mm-wave frequencies at a center frequency of 30 GHz, which can be deployed for the next generation of mobile communication. The designed coupler provides a wide matching and isolation bandwidth with low output amplitude imbalance, which is unique in comparison with current couplers. The prototype of the proposed coupler is fabricated and measured where the simulation and measurement results show a good agreement indicating the strength of the proposed method in PRGW structure design as well. The measured results show the couplers achieve better than 10-dB return loss and isolation over the frequency range from 25 to 40 GHz (46% BW) with the power-split unbalance and phase error within ± 1 dB and ± 5°, respectively. In addition, square mushrooms are chosen here to satisfy the high impedance surface. Not only do they bring about larger stop bandwidth, but also their configuration facilitates the arrangement of them around the coupler. The proposed design has superb characteristics such as low profile, low loss, and easy integration with microwave circuits and systems that can be suitable for designing mm-wave beamforming networks.

## Introduction

Upcoming sixth-generation (6G) and fifth-generation (5G) wireless communication technology have fundamentally revolutionized the telecommunications industry^1^. The next generation of mobile communications requires the use of a high-frequency spectrum due to the limited channel bandwidth of the current ones which have worked in microwave frequency channels^2^. Millimeter-wave (mm-wave) frequency band starting from 30 to 300 GHz is a good candidate for the proposed purpose. mm-wave spectrum with massive available bandwidth is a promising technology for the next generation to boost the data-rate transmission on the order of multigigabit/s and triumph the bandwidth shortage at saturated traditional microwave spectrum^3,4^. This evolution of wireless data communications from today's microwave and lower frequency bands to mm-wave bands has created challenges and opportunities for mobile service designers^5–7^.

In this context, a lot of works have been carried out regarding the design of couplers, antennas, filters, and resonators^8–14^. The technologies used for designing the components in mm-wave frequency bands have been mostly microstrip lines, waveguides, and Substrate Integrated Waveguides (SIW)^15–17^. However, there exists a big problem associated with these technologies, being lossy owing to dielectric and conductor losses or leakage from via including walls^18–21^. To that end, recently, a new technology called Ridge Gap Waveguide (RGW) or particularly Printed-RGW (PRGW) structures are considered to solve this problem^22–28^, this enables the electromagnetic wave to propagate in the air gap between the conductor and ridge and consequently eliminates the dielectric losses. In addition, the leakage of energy reduces as a result of the bed of nails in RGW or Electromagnetic Band Gaps (EBGs) in PRGW^29^.

On the other hand, the realization of the 5G communication system at the mm-wave frequency with short wavelengths is limited by high path loss and atmospheric absorption, implying a reduced communication range. Although this limitation can be compensated using high-gain antennas, they have a directional narrow beamwidth requiring beam-switching techniques to compensate for the main beam misalignment. Beam switching networks are necessary to address the challenges and expectations of future technology. These can be summarized as high-power efficiency, multiuser systems, and large channel capacity with wide scanning coverage.

Butler Matrix (BM) as a Beam-switching feed network with its various modifications can satisfy the mentioned goal^30–34^. BM includes couplers, crossovers, and power dividers, which should be designed and then arranged in a specific way. There have been some publications regarding PRGW and RGW couplers^35^. In^36^, the already existing branch line coupler and coupled line directional coupler^37^, have been implemented by means of the bed of nails and ridge configuring the couplers. Although the results are satisfactory, the fabrication process is challenging in this way. Authors in^38,39^ have investigated directional couplers on a silicon platform based on coupled-mode theory. Although they can be used in mm-wave frequencies however they have weak coupling and there are no phase difference that limit their application.

In^40^, the printed-RGW coupler with a novel structure has been introduced, which provides a good impedance bandwidth as well as a little output phase and amplitude imbalance. It should be mentioned that as it is impossible to directly connect connectors to the structure, a microstrip transition line must be used which is described in this paper as well. Another similar structure with mathematical equations, which enables the structure more flexible in other mm-wave frequency bands, has been proposed in^41^. Authors in^42,43^ have designed low-profile hybrid directional couplers for 5G communication purposes. Furthermore, a Rat-Race coupler, which has the ability to divide the input power with 0° or 180° phase difference has been implemented in^44^ by means of PRGW technology.

However, mentioned couplers own a wide impedance bandwidth, and they suffer from output amplitude and phase imbalance, which makes the whole structure narrowband, so it is not possible to design a broadband feeding network in mm-wave frequencies including directional couplers. Also, their simulation process is tough as a result of EBG unit cells placed around the structure to act as high impedance surface avoiding surface wave propagation. Thus, a suitable boundary condition is required to make the design process easier. There is no need to put the EBG unit cells in primary structure in this situation. Getting the required results, for the sake of final optimization, the complete coupler with high impedance surfaces is simulated. The proposed procedure reduces the simulation time and results in the desired performance that is difficult to obtain from the full optimization design process. Using this technique, an ultra-wideband PRGW 3 dB hybrid coupler is designed to obtain a 46% bandwidth of reflection coefficient as well as isolation below − 10 dB over the frequency range from 25 to 40 GHz.

The superb results are achieved as a result of the proposed design procedure that not only facilitates the design by avoiding the EBG in the design phase but also allows to achieve very good performance by eliminating the positioning of the EBGs structure and the nails in the ridge. These structures dramatically increase the simulation time as the mesh size is significantly increased. The proposed design procedure is applied to design a coupler, however, it can be also applied to design large structures and save a huge time and effort. The novelty of our paper not only is to introduce the new designing RGWs structures method but also to design a wideband coupler that has the best performance in comparison with similar works in mm-wave frequencies.

## PRGW double-box hybrid coupler

In this section, a step-by-step design procedure of the wideband hybrid coupler based on PRGW technology is illustrated. At the first place, the periodic square EBG unit cell surrounding the ridge is designed with the proper dimensions to suppress any leakage and generate a wide bandgap range from 24 to 45 GHz required for the 5G applications. A boundary condition with regard to the obtained air gap height between EBG unit cells and the upper conductor is defined to help facilitate the design of the primary coupler. The traditional branch-line coupler is designed by the proposed boundary condition to validate the robustness of the method. Subsequently, the broadband double-box hybrid coupler with the same proposed boundary condition is simulated. Upon getting the desired results, EBG unit cells are applied to the structure and a final optimization is performed. Finally, the parameters of the fabricated structure are evaluated and compared with simulated results.

### Design of EBG unit cell and PRGW line

The ideal RGW utilizes the basic cutoff band related to the perfect electrical conductor (PEC) and perfect magnetic conductor (PMC) parallel plate waveguide configuration. There is no propagated field in the air gap between the PEC surface and the PMC surface as long as the spacing between the two surfaces, referred to as the gap height (H), is less than a quarter of a wavelength (λ/4).

**Figure 1:**
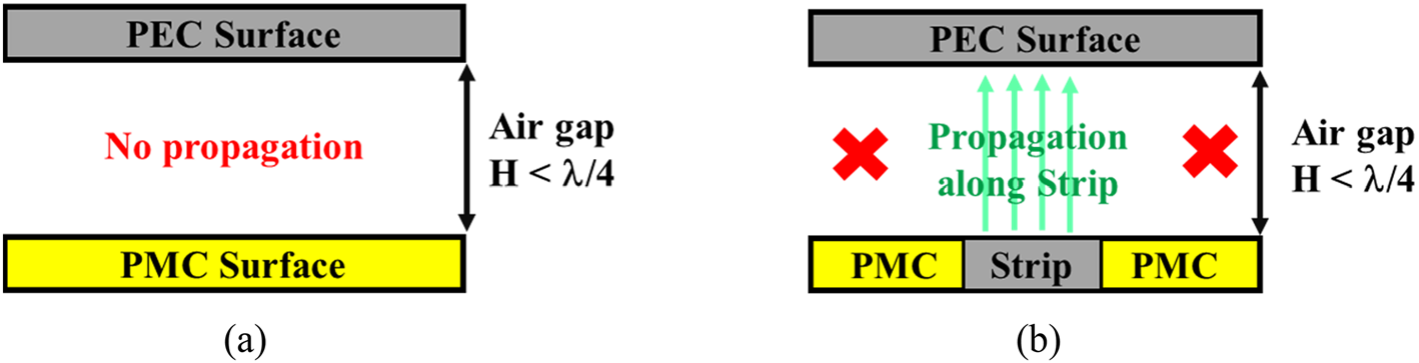
Field propagation (**a**) within two parallel plates consists of a PEC plate and PMC plate, and (**b**) a single-texture side of ideal RGW.

In Fig. 1, the ideal form of the proposed concept is shown, whereas it is obvious in RGW, a metal strip or specifically the ridge is surrounded by PMC surfaces. Providing a waveguide cavity height smaller than λ/4, the electromagnetic wave (EM) can propagate between the upper conductor and ridge as PEC-PEC surfaces suppress the leakage in all directions as PMC-PEC surfaces.

**Figure 2:**
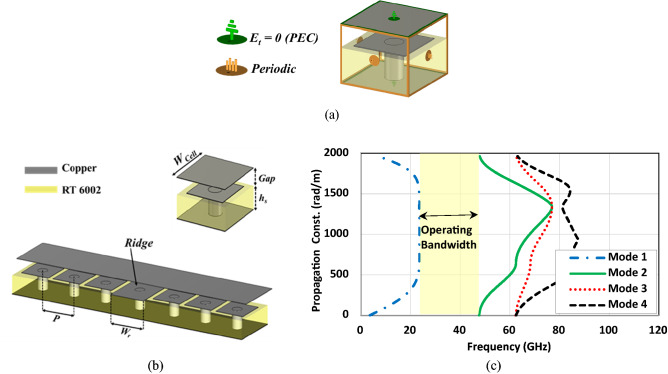
PRGW structure design. (**a**) The periodic boundary condition of designing EBG unit cell in CST Microwave Studio. (**b**) Section of PRGW guiding structure, and (**c**) dispersion diagram of the square PRGW unit cell (W_cell_ = 1.6, Gap = 0.254, h_s_ = 0.762, W_r_ = 1.34, P = 1.4 (all in mm)).

However, since PMC does not exist in nature, it is realized by an artificial magnetic conductor (AMC) that mimics the PMC behavior over a specific bandwidth^45^. An EBG unit cell is an AMC that can satisfy the mentioned characteristics, so a period of them is used in PRGW structures, which are a printed modified shape of RGW. This periodic part is used to block the electromagnetic waves from leakage outside the region between the ridge and upper conductor within a certain stopband. Figure 2 depicts the EBG unit cell and its dispersion diagram over a wide frequency range that is obtained using the computer simulation technology (CST) (Eigen-mode solver). Periodic boundary conditions are used to model the whole EBG unit cell structure. Eigen-mode solver parameter sweep is used to step through the phase assigned to the periodic boundaries without using any ports^40,46^. As in the frequency gap from 24 to 45 GHz, the propagation constant value is zero, it is concluded that in the mentioned frequency range, an EBG unit cell acts as PMC, thus resulting in propagation suppression. The height of the air-filled gap between the square-grounded patch and the upper conductor plays a key role in defining the perfect boundary condition. It is also worth mentioning that the substrate used here is Rogers RT 6002 with the dielectric constant of 2.94 and a height of 0.762 mm. The patch is square with a length and width of 1.2 mm and the air-filled region has a height of 0.254 mm.

### Design of the primary single-box branch line coupler

**Figure 3:**
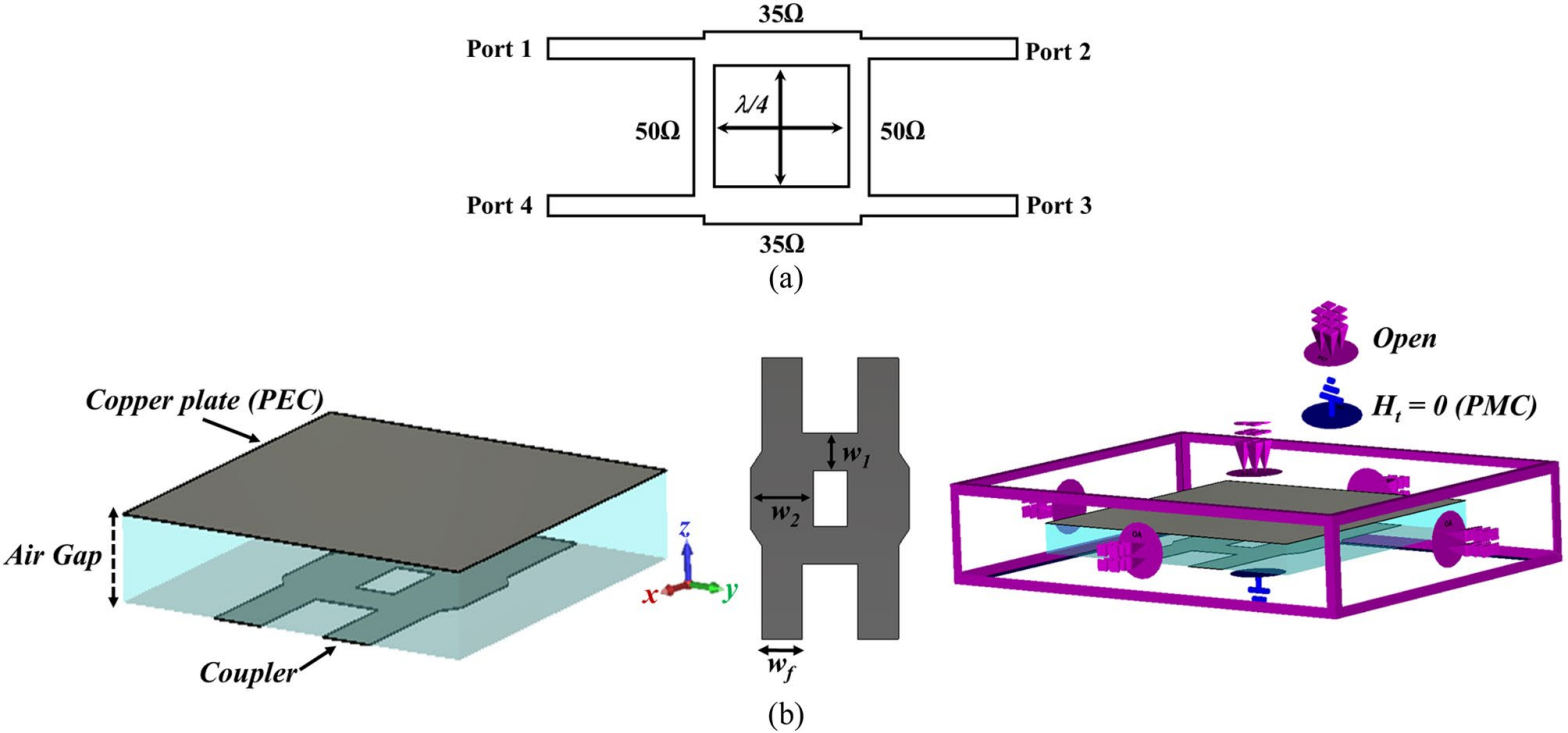
Single-box branch line coupler. (**a**) Basic schematic, (**b**) the specific boundary condition in CST Microwave Studio (W_1_ = 1.3, W_2_ = 2.1, W_f_ = 1.34 (all in mm)).

**Figure 4:**
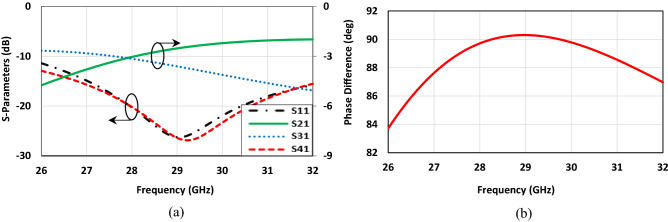
Simulated results of the Single-box branch line coupler with perfect boundary condition. (**a**) Scattering parameters, (**b**) phase difference between output ports (Φ (S_21_)–Φ (S_31_)).

**Figure 5:**
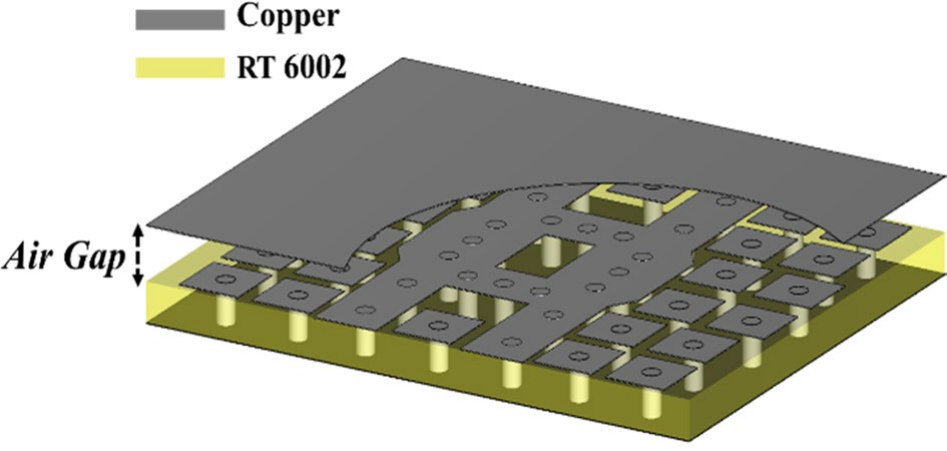
The configuration of the traditional PRGW branch-line coupler.

**Figure 6:**
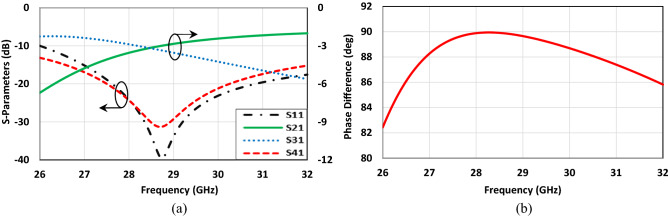
Simulated results of the Single-box branch coupler with EBG unit cells. (**a**) Scattering parameters, (**b**) phase difference between output ports (Φ (S_21_)–Φ (S_31_)).

To illustrate the specific boundary condition, the traditional single-box branch-line coupler is proposed^47,48^. Figure 3 shows the configuration of the coupler operating at 28 GHz in CST Microwave Studio. The structure only consists of the branch-line coupler patch without the EBG unit cells, and a metal plate as a PEC surface placed above it. The air-filled gap between the coupler patch and PEC is already determined in the unit cell design process, which is obtained using CST software (Eigen-mode solver) from the previous section. When it comes to talking about the boundary condition, it should be mentioned that it is required to define the PMC condition, (H_t_ = 0), instead of using EBG periodic structure at Z_min_ = 0 for the plane touching the coupler patch and assign the open boundary condition for other faces, see Fig. 3b. As it is evident the configuration is so simple, and it takes a few minutes to observe the results. However, it is not the case as one desires to follow the already existing design method. Figure 4 depicts the scattering parameters of the coupler. The amplitude imbalance in the region between 27 to 29 GHz is acceptable making the structure so narrow band. It should also be pointed out that the phase difference imbalance is acceptable in the entire frequency band. In the following step, the designed EBG unit cells are placed surrounding the coupler, and the branch itself is grounded by means of a series of vias, Fig. 5. All the parameters and lengths are almost the same as Fig. 1 for unit cells and Fig. 3 for the coupler. The distance between the mushrooms is 0.2 mm as well. Figure 6 depicts the results achieved upon applying the EBG unit cells around the branch-line coupler.

It is evident from Fig. 6a that the results are completely in agreement with those obtained from the coupler with specific boundary conditions. From the frequency range of 27–29 GHz, the reflection coefficient and isolation are almost acceptable as the imbalance between the amplitude output signals is satisfactory. Furthermore, it is observed from Fig. 6b that the phase difference between output signals is undoubtedly so close to each other in both conditions; the imbalance is less than 2°. Overall, one can conclude that the presented procedure is a powerful tool in designing the structures based on PRGW technology.

### Design of the broadband coupler with specific boundary conditions

**Figure 7:**
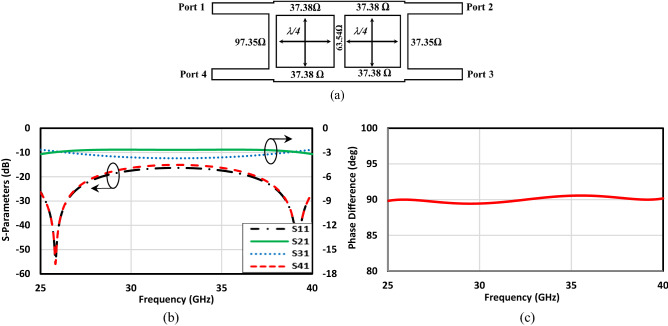
Double-box ideal hybrid coupler. (**a**) Basic schematic, (**b**) scattering parameters, (**c**) phase difference between output ports (Φ (S_21_)–Φ (S_31_)).

**Figure 8:**
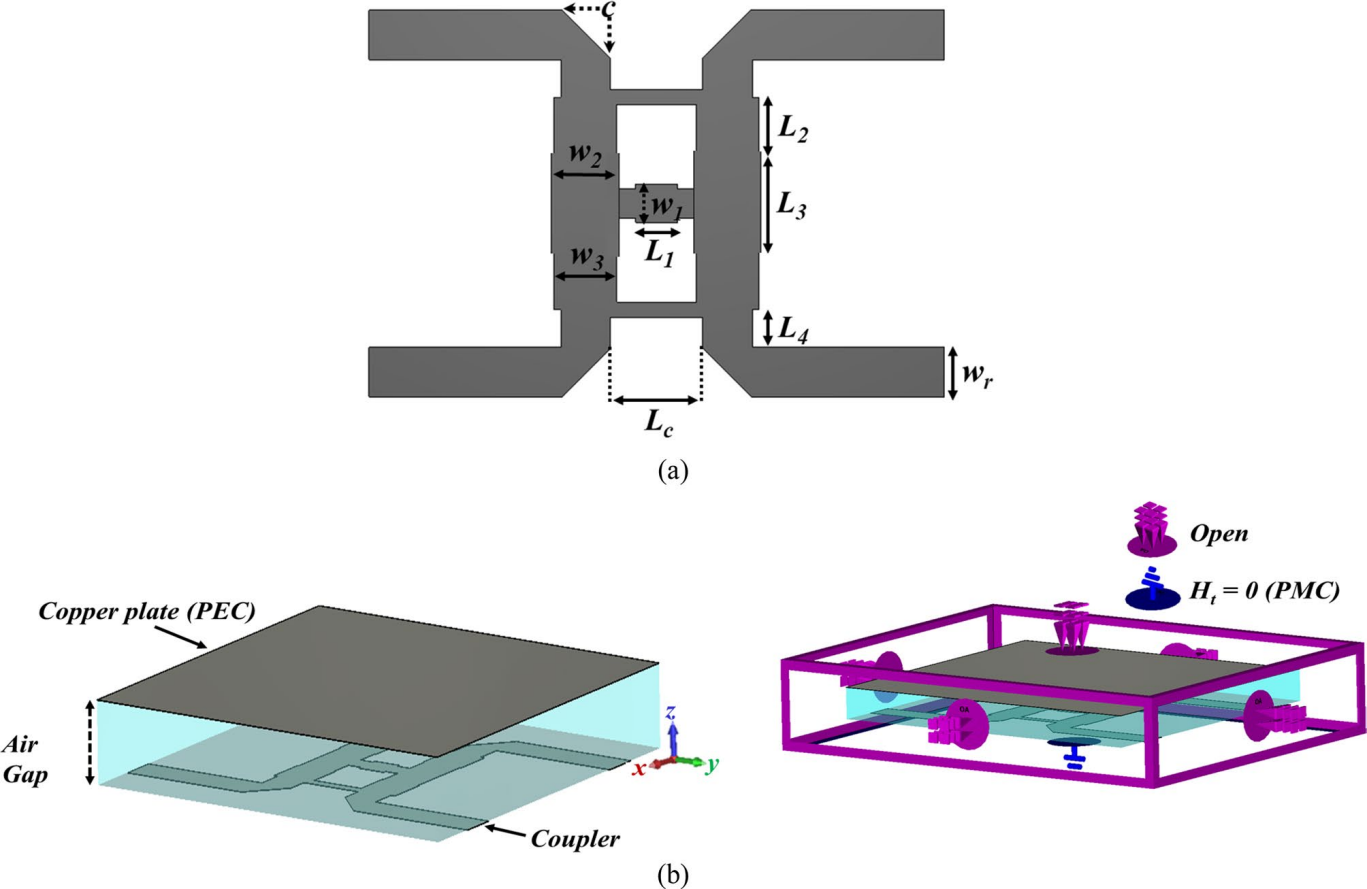
(**a**) The geometrical configuration of the double-box hybrid coupler, (**b**) the specific boundary condition in CST Microwave Studio (c = 1.3, W_1_ = 1.02, W_2_ = 1.67, W_3_ = 1.62, W_r_ = 1.34, L_1_ = 1.12, L_2_ = 1.54, L_3_ = 2.62, L_4_ = 1, L_c_ = 2.46 (all in mm)).

**Figure 9:**
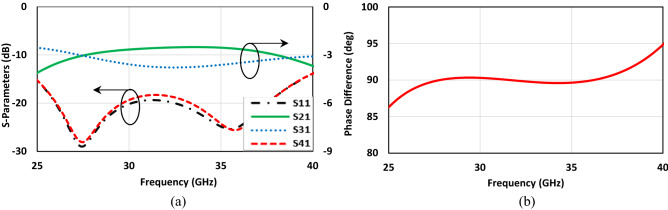
Simulated results of the double-box coupler with specific boundary condition. (**a**) Scattering parameters, (**b**) phase difference between output ports (Φ (S_21_)–Φ (S_31_)).

Traditional branch line coupler gives rise to a narrowband structure, as the imbalance between output amplitudes gets larger and larger when a broad frequency range is considered. This disturbs the performance of the system in which the coupler is going to be embedded. As an illustration, to use the coupler in a broadband Butler Matrix networks, there would be some issues with the narrowband coupler. Thus, to improve the bandwidth, one way is to add another section following the branch line as dual-box 3 dB coupler. By so doing, the characteristic impedance of arms is changed; however, their length remains quadrature-wavelength^48^. Figure 7a shows the schematic of the proposed structure, Fig. 7b,c depict the transition as well as the reflection coefficients and the phase difference of the structure in the ideal situation, respectively. The results are completely satisfactory due to the fact that the amplitude imbalance is small and acceptable over the large frequency band. This has its root in the second box that operates at a frequency close to the operation of the first box and consequently makes the structure broadband. In the next step, we use a full-wave solver tool, CST Microwave Suit, to simulate the structure with the specific boundary condition, which helps achieve the desired results without involving mushroom unit cells. Figure 8 depicts the coupler in the proposed boundary condition that enables one to reach the simulated results as quickly as possible. Figure 9 demonstrates the scattering parameters of the broadband coupler considering the boundary condition. It should be mentioned that from the frequency range of 25 GHz up to 40 GHz the output amplitude imbalance is near ± 1 dB and the phase difference between output ports is between ± 5°. These characteristics are unique in comparison with current mm-wave hybrid couplers.

### Design and optimization of the broadband PRGW coupler

**Figure 10:**
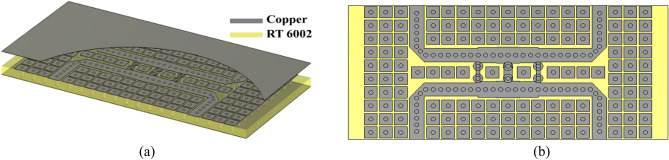
(**a**) Block diagram of the broadband hybrid coupler, (**b**) 3-D view, (**b**) top view (upper ground is removed for clear illustration).

**Figure 11:**
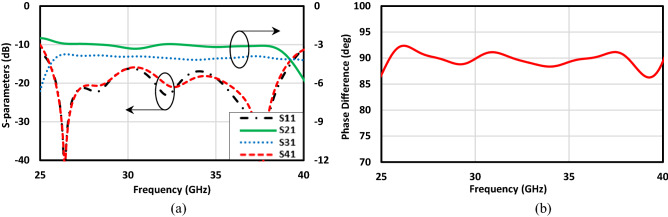
Simulated results of the broadband PRGW coupler. (**a**) Scattering parameters, (**b**) phase difference between output ports (Φ (S_21_)–Φ (S_31_)).

After designing the broadband hybrid coupler with the aid of specific boundary conditions, it is time to apply EBG unit cells as shown in Fig. 10. The S-parameter and phase difference between the output ports of the broadband coupler are depicted in Fig. 11. It is obvious from Fig. 11a that the − 10 dB impedance bandwidth of the structure is from 25 to 40 GHz, and the input signal is almost divided into two equal signals and received from output ports over this frequency band. Referring to the operation of the unit cell, it is concluded that the whole stop band area of the unit cell is well utilized. However, in case the traditional circular EBG unit cells are used, such bandwidth is not achievable. Also, it is evident from Fig. 11b that the phase difference between output signals is roughly 90° over a wide frequency range. It is worth mentioning that the phase imbalance is near ± 5° and the amplitude imbalance is around 1 dB. Thus, we can assert that by means of the proposed boundary condition, it is the simplest way to optimize the structure without any mushroom unit cells and use the achieved parameters including the size of arms and their length to reach the final PRGW coupler.

Although the results of the double coupler structure with perfect boundary conditions and the structure with EBG unit cells are close, the results of the double coupler are more different from the perfect one compared to the single coupler. This difference is because the double-box coupler structure is more complex than the single coupler and the number of EBG unit cells used in double-box coupler are more than the single coupler.

## Design of microstrip to PRGW transition

**Figure 12:**
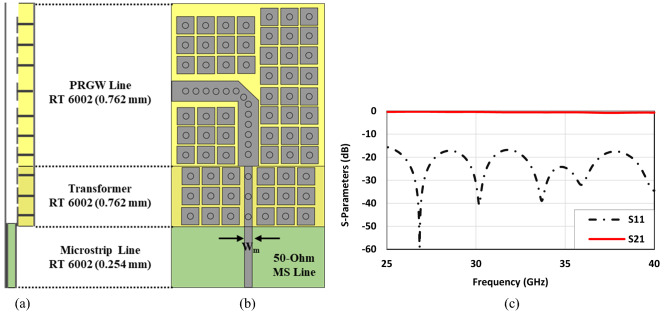
Block diagram of 90° bend PRGW with microstrip to PRGW transition. (**a**) Side view, (**b**) top view, (**c**) scattering parameters (W_m_ = 0.633 mm).

As it was mentioned before, it is not possible to directly connect SMA connectors to the PRGW structures for measurement purposes, so it needs a transition line from microstrip line to PRGW. The schematic of the 90° bend transition is depicted in Fig. 12a,b. Rogers RT 6002 with the thickness equivalent to the required gap height between unit cells and top conductor is utilized here to provide a transmission line with the characteristic impedance of 50-Ω. Subsequently, this microstrip line is connected to its PRGW counterpart and finally the transition is performed. One important point which should be considered is related to the fact that the reflection coefficient of the transition line must be below − 10 dB and its transition coefficient should be about 0 dB over the operation frequency band. To validate the performance of the transmission line, it is designed and simulated separately. Figure 12c illustrates the s-parameters of the structure. Over the whole frequency band from 25 to 40 GHz, the signal is transferred from Port 1 to Port 2 with a minimum reflection and maximum transition coefficient. Such a transmission line is integrated with the PRGW coupler as shown in Fig. 12b, which will be demonstrated in the following section. Furthermore, it should be taken into consideration that the EBG unit cells used for the line are the same as those used for the main structure.

## Experimental validation

**Figure 13:**
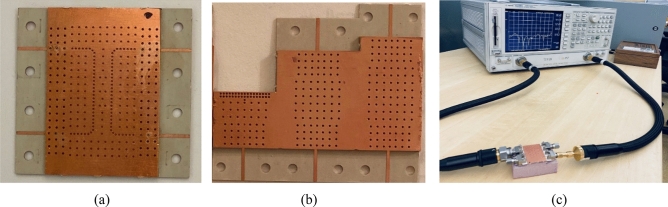
(**a**) Fabrication of the proposed broadband PRGW coupler, (**b**) TRL calibration kit, (**c**) measurement setup.

**Figure 14:**
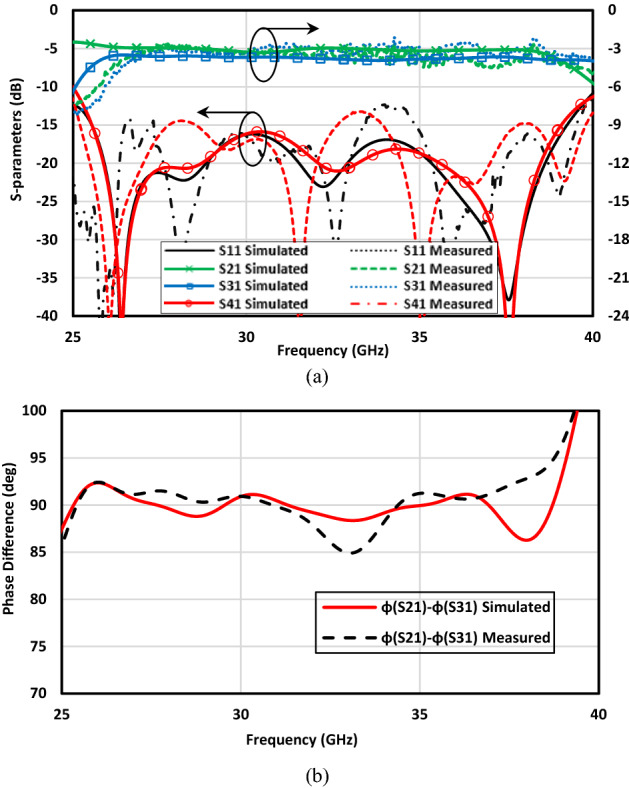
Simulated and measured results of the proposed broadband PRGW coupler. (**a**) Scattering parameters, (**b**) phase difference between output ports (Φ (S_21_)–Φ (S_31_)).

Having designed the broadband coupler, it is fabricated and measured to validate the results achieved from simulations, see Fig. 13a. The 2.92 mm End launch SMA connectors are used in the measurement setup. Our expectation is to obtain a reflection coefficient as well as isolation below − 10 dB over the frequency range from 24 to 40 GHz as achieved in performed simulations. Also, it is our favorite to have two equal signals with a ± 90° phase difference according to the exciting input port. However, as mentioned before, an amplitude imbalance of 1 dB and phase imbalance of ± 5° are acceptable for many applications. With all this in mind, the measurement setup shown in Fig. 13c is taken place. Since the coupler is totally symmetric, there is no need to measure the scattering parameters for both input ports. Moreover, a TRL (Thru-Reflect-Line) calibration kit depicted in Fig. 13b is fabricated to calibrate the network analyzer locally. The reflect or short, thru, and line circuit required for calibration of any available network analyzer is integrated into one package. It is also obvious that the transmission lines designed in the previous section are fully integrated with the PRGW coupler as well as the TRL calibration kit. The measured results are presented in Fig. 14. The impedance bandwidth covers the whole frequency range shown in Fig. 14a. However, there are some differences between the simulated and measured results. This holds true as a result of minor fabrication errors and unavoidable connection problems. When it comes to talking about the output amplitudes and phases, it can be easily observed that the desired results are obtained. From the frequency of 25 GHz up to 39 GHz the defined imbalances are considered. Thus, it can be concluded that the proposed method with the aid of specific boundary conditions in designing the PRGW structures is reliable and accelerates the design process.

## PRGW 3-dB hybrid coupler performance evaluation

In order to evaluate the performance of the coupler and compare it with existing works, Table 1 is provided where the focus is on the guiding structures, as they are the promising technologies for the mm-wave applications. Most of the 3 dB hybrid couplers presented in the literature have a narrow bandwidth of up to 18% and high output phase and amplitude imbalance^40,43,49–52^. The offered couplers in^41,42^ have a wider impedance bandwidth of 26.5% and 26.6% respectively, as well as a low output phase and amplitude imbalance while they suffer from bigger size. The authors in^53^ have designed a SIGW hybrid coupler that provides 26.4% bandwidth. However, it has a weak amplitude and phase balance. The proposed PRGW coupler size is 1.1λ_g_ × 0.75λ_g_ at the frequency center of 30 GHz with the isolation and reflection loss below − 10 dB over the whole frequency band from 25 to 40 GHz. Considering the frequency band in which the output imbalance is below 1 dB, the proposed coupler provides the widest bandwidth. In addition, the proposed coupler provides 43% phase balance bandwidth with an imbalance between 90° ± 5°, namely from 25 to 39 GHz. The results are indicative of the fact that the hybrid coupler suggested in this paper has the best performance in comparison with similar works implemented with state of art guiding structure technologies such as PRGW, RGW, and SIW, in mm-wave frequencies.

**Table 1:** Performance comparison of the presented PRGW 3 dB hybrid coupler with other proposed couplers in the literature.

Ref	Technology	Center frequency (GHz)	Bandwidth (S_11_ < − 10 dB)	Amplitude balance BW (dB)	Phase impedance BW %	Size (λ_g_ × λ_g_)
^40^	PRGW	30	6%	− 3.6 ± 1 BW = 6%	90° ± 10 BW = 6%	1.1 × 1.1
^41^	PRGW	30	26.5%	− 3.7 ± 0.75 BW = 6%	90° ± 5 BW = 6%	1.3 × 1.3
^42^	PRGW	30	26.6%	− 3.6 ± 0.75 BW = 13%	90° ± 5 BW = 26.5%	1.2 × 1.2
^43^	PRGW	30	13%	− 3.6 ± 0.5 BW = 6.7%	90° ± 10 BW = 14%	1.2 × 1.2
^49^	Microstrip	30	11%	− 4 ± 1 BW = 11%	90° ± 1 BW = 11%	1.3 × 1.1
^50^	SIW	24	18%	− 4.7 ± 0.5 BW = 10%	92 ± 2 BW = 18%	1.4 × 1.5
^51^	RGW	15	14%	− 3.5 ± 0.75 BW = 7%	–	1.58 × 1.58
^52^	PRGW	60	11%	− 3.7 ± 0.5 BW = 11%	90° ± 5 BW = 11%	1.9 × 1.1
^53^	SIGW	26	26.4%	− 3.7 ± 1 –	90° ± 10 –	1 × 1
This work	PRGW	30	46%	− 3.5 ± 0.5 BW = 41%	90° ± 5 BW = 43.75	1.1 × 0.75

## Conclusion

In this paper, a specific boundary condition has been presented to facilitate the design of PRGW structures. Taking advantage of the proposed boundary condition, a wideband dual-box hybrid coupler has been designed, fabricated, and measured. The reflection loss and isolation between two adjacent ports of the coupler is below − 10 dB of 46% in the frequency range from 25 to 40 GHz. In addition, the band in which the output amplitude imbalance is less than 1 dB is from 26 to 39 GHz. This holds true for the band in which the output phase imbalance is between ± 5̊. The comparison between this work and already existing counterparts shows that the proposed coupler has unprecedented characteristics that enable it to be used in feeding networks requiring wideband components.

## Data Availability

The datasets used and/or analyzed during the current study are available from the corresponding author on reasonable request. Zahra Mousavirazi (Zahra.mousavirazi@inrs.ca).

## References

[1] Lak A, Adelpour Z, Oraizi H, Parhizgar N (2021). Design and SAR assessment of three compact 5G antenna arrays. Sci. Rep. UK.

[2] Ali, M. M. M. & Sebak, A. R. Directive antennas for future 5G mobile wireless communications. *2017 XXXIIND General Assembly and Scientific Symposium of the International Union of Radio Science *(*URSI GASS*) (2017).

[3] Razi ZM, Rezaei P, Valizade A (2015). A novel design of Fabry–Perot antenna using metamaterial superstrate for gain and bandwidth enhancement. AEU Int. J. Electron. Commun..

[4] Elsaadany M, Ali MMM, Shams SI, Denidni TA, Gagnon G (2021). A novel design technique for mm-wave mismatch terminations. IEEE Trans. Microw. Theory Tech..

[5] Mousavirazi Z, Rafiei V, Ali MMM, Denidni TA (2021). Wideband and low-loss magneto-electric dipole antenna fed by printed ridge-gap waveguide technology. Int. Symp. Antenna Technol..

[6] Mousavirazi Z, Denidni TA (2021). A circularly-polarized antenna for 5G applications fed by printed ridge-gap waveguide. Int. Symp. Antenna Technol..

[7] Mousavirazi, Z., Tuloti, S. H. R. & Denidni, T. A. A millimeter-wave low-profile and metal-only transmitarray antennas at 28 GHz. *2020 14th European Conference on Antennas and Propagation *(*EUCAP 2020*) (2020).

[8] Mousavi Z, Rezaei P (2019). Millimetre-wave beam-steering array antenna by emphasising on improvement of Butler matrix features. IET Microw. Antennas Propag..

[9] Tuloti SHR, Mousavirazi Z, Kesavan A, Denidni TA (2022). A low profile dual-polarized transmitarray antenna at Ka-band. AEU-Int. J. Electron. Commun..

[10] Ali MMM, Sebak AR (2018). 2-D scanning magnetoelectric dipole antenna array fed by RGW butler matrix. IEEE Trans. Antennas Propag..

[11] Afifi I, Ali MMM, Sebak AR (2018). Analysis and design of a wideband coaxial transition to metal and printed ridge gap waveguide. IEEE Access.

[12] Sifat SM, Ali MMM, Shams SI, Sebak AR (2019). High gain bow-tie slot antenna array loaded with grooves based on printed ridge gap waveguide technology. IEEE Access.

[13] Ali MMM, Afifi I, Sebak AR (2020). A dual-polarized magneto-electric dipole antenna based on printed ridge gap waveguide technology. IEEE Trans. Antennas Propag..

[14] Ali MMM, Shams SI, Sebak AR (2018). Low loss and ultra flat rectangular waveguide harmonic coupler. IEEE Access.

[15] Diman AA (2021). Efficient SIW-feed network suppressing mutual coupling of slot antenna array. IEEE Trans. Antennas Propag..

[16] Mousavirazi, Z., Rafiei, V., Ali, M. M. M. & Denidni, T. A. A Wideband CP cavity-backed SIW antenna fed by printed-RGW technology. *2021 IEEE International Symposium on Antennas and Propagation and USNC-URSI Radio Science Meeting *(*APS/URSI*), 1531–1532. 10.1109/APS/URSI47566.2021.9704236 (2021).

[17] Alibakhshikenari M (2021). High-isolation antenna array using SIW and realized with a graphene layer for sub-terahertz wireless applications. Sci. Rep. UK.

[18] Hitzler, M., Iberle, J., Mayer, W., Barth, H. & Waldschmidt, C. Wideband low-cost hybrid coupler for mm-wave frequencies. *2017 IEEE MTT-S International Microwave Symposium *(*IMS*), 626–629 (2017).

[19] Kesavan A, Al-Hassan M, Ben Mabrouk I, Denidni TA (2021). Wideband circular polarized dielectric resonator antenna array for millimeter-wave applications. Sensors..

[20] Park SJ, Park SO (2017). LHCP and RHCP substrate integrated waveguide antenna arrays for millimeter-wave applications. IEEE Antennas Wirel. Propag..

[21] Jankovic, U., Mohottige, N., Budimir, D. & Glubokov, O. Hybrid manufactured waveguide resonators and filters for mm-wave applications. *2017 IEEE MTT-S International Microwave Workshop Series on Advanced Materials and Processes for RF and THz Applications* (*IMWS-AMP*) (2017).

[22] Alfonso, E., Kildal, P. S., Valero-Nogueira, A. & Baquero, M. Study of the characteristic impedance of a ridge gap waveguide. *IEEE Antennas Propag.*, 2935 (2009).

[23] Sorkherizi MS, Dadgarpour A, Kishk AA (2017). Planar high-efficiency antenna array using new printed ridge gap waveguide technology. IEEE Trans. Antennas Propag..

[24] Rahiminejad S (2012). Micromachined ridge gap waveguide and resonator for millimeter-wave applications. Sensors Actuators A Phys..

[25] Sorkherizi MS, Kishk AA (2016). Fully printed gap waveguide with facilitated design properties. IEEE Microw. Wirel. Compon. Lett..

[26] Shi S, Qian ZP, Ding WP (2019). Wideband circularly polarized leaky-wave antenna with flat gain using printed ridge gap waveguide. Int. J. Rf Microw. C E.

[27] Mousavirazi, Z., Rafiei, V., Ali, M. M. M. & Denidni, T. A. A high-efficiency zeroth-order resonant (ZOR) antenna fed by printed ridge-gap waveguide technology. *2021 International Applied Computational Electromagnetics Society Symposium *(*ACES*). 10.1109/Aces53325.2021.00109 (2021).

[28] Mousavirazi, Z., Akbari, M. & Denidni, T. A. Millimeter-wave high-gain PRGW antenna using a Fabry-Perot cavity. *IEEE Antennas Propag.*, 1365–1366. 10.1109/Ieeeconf35879.2020.9329905 (2020).

[29] Al-Alem Y, Sifat SM, Antar YMM, Kishk AA (2021). High gain low-cost 20 GHz antenna design based on the utilization of diffracted fields from dielectric edges. Int. Symp. Antenna Technol..

[30] Afifi I, Sebak AR (2020). Wideband 4 × 4 Butler matrix in the printed ridge gap waveguide technology for millimeter-wave applications. IEEE Trans. Antennas Propag..

[31] Mousavi Z, Rezaei P, Kakhki MB, Denidni TA (2019). Beam-steering antenna array based on a butler matrix feed network with CP capability for satellite application. J. Instrum..

[32] Nasseri H, Bemani M, Ghaffarlou A (2020). A new method for arbitrary amplitude distribution generation in 4 × 8 Butler matrix. IEEE Microw. Wirel. Compon. Lett..

[33] Mousavirazi Z, Rafiei V, Denidni TA (2021). Beam-switching antenna array with dual-circular-polarized operation for WiMAX applications. AEU-Int. J. Electron. C.

[34] Razi ZM, Rezaei P (2020). A two-layer beam-steering array antenna with 4 × 4 modified Butler matrix fed network for switched beam application. Int. J. Rf Microw. C E.

[35] Mousavirazi, Z., Ali, M. M. M. & Denidni, T. A. *2021 IEEE International Symposium on Antennas and Propagation and USNC-URSI Radio Science Meeting* (*APS/URSI*) 1063–1064 (2021).

[36] Alfonso, E. *et al.* Design of microwave circuits in ridge-gap waveguide technology. *IEEE MTT S Int. Microw. Symp.*, 1544–1547 (2010).

[37] Pozar DM (1990). A modern course in microwave engineering. IEEE Trans. Educ..

[38] Mao D (2019). Adiabatic coupler with design-intended splitting ratio. J. Lightw. Technol..

[39] Chen L, Li X, Gao D (2012). An efficient directional coupling from dielectric waveguide to hybrid long-range plasmonic waveguide on a silicon platform. Appl. Phys. B.

[40] Ali MMM, Shams SI, Sebak A-R (2018). Printed ridge gap waveguide 3-dB coupler: Analysis and design procedure. IEEE Access.

[41] Ali MMM, Shams SI, Sebak A (2019). Ultra-wideband printed ridge gap waveguide hybrid directional coupler for millimetre wave applications. IET Microw. Antennas Prorag..

[42] Ali MMM (2021). A systematic design of a compact wideband hybrid directional coupler based on printed RGW technology. IEEE Access.

[43] Zhao Z, Denidni TA (2020). Millimeter-wave printed-RGW hybrid coupler with symmetrical square feed. IEEE Microw. Wirel. Compon. Lett..

[44] Afifi I, Sebak AR (2020). Wideband printed ridge gap rat-race coupler for differential feeding antenna. IEEE Access.

[45] Bayat-Makou N, Kishk AA (2018). Realistic air-filled TEM printed parallel-plate waveguide based on ridge gap waveguide. IEEE Trans. Microw. Theory Tech..

[46] Razavi SA, Kildal PS, Xiang LL, Alos EA, Chen HG (2014). 2×2-Slot element for 60-GHz planar array antenna realized on two doubled-sided PCBs using SIW cavity and EBG-type soft surface fed by microstrip-ridge gap waveguide. IEEE Trans. Antennas Propag..

[47] Gupta RK, Anderson SE, Getsinger WJ (1987). Impedance-transforming 3-db 90-degrees hybrids. IEEE Trans. Microw. Theory Tech..

[48] Grebennikov A (2011). RF and Microwave Transmitter Design.

[49] Ye XF, Zheng SY, Pan YM (2016). A compact millimeter-wave patch quadrature coupler with a wide range of coupling coefficients. IEEE Microw. Wirel. Compon. Lett..

[50] Djerafi T, Wu K (2007). Super-compact substrate integrated waveguide cruciform directional coupler. IEEE Microw. Wirel. Compon. Lett..

[51] Shams SI, Kishk AA (2017). Design of 3-dB hybrid coupler based on RGW technology. IEEE Trans. Microw. Theory Tech..

[52] Farahani M, Akbari M, Nedil M, Denidni TA, Sebak AR (2017). A novel low-loss millimeter-wave 3-dB 90° ridge-gap coupler using large aperture progressive phase compensation. IEEE Access.

[53] Shen D, Wang K, Zhang X (2018). A substrate integrated gap waveguide based wideband 3-dB coupler for 5G applications. IEEE Access.

